# Investigating the social environment of the A‐not‐B search task

**DOI:** 10.1111/desc.12921

**Published:** 2019-11-26

**Authors:** Kirsty Dunn, James Gavin Bremner

**Affiliations:** ^1^ Lancaster University Lancaster UK

**Keywords:** cognitive development, infant behaviour, object permanence, social cues, social looking, violation of expectation

## Abstract

Controversy exists concerning the origins of object permanence, with different measures suggesting different conclusions. Looking measures have been interpreted as evidence for early understanding (Baillargeon, 1987, *Developmental Psychology*, **23**:655), while Piaget (*The construction of reality in the child*, 1954) interpreted perseverative reaching behaviour on his AB search task to be indicative of limited understanding. However, looking measures are often reported to be an unreliable index of infant expectation (Haith, 1998, *Infant Behaviour and Development*, **21**:167) and reaching behaviour has been explained by many alternative processes (e.g. Smith et al., 1999, *Psychological Review*, **106**:235; Topál et al., 2008, *Science*, **321**:1831). We aimed to investigate whether social looking (Dunn & Bremner, 2017, *Developmental Science*, **20**:e12452; Walden et al., 2007, *Developmental Science*, **10**:654) can be used as a valid measure of infant expectation of object location during the Piagetian AB search task. Furthermore, we aimed to test the social accounts of perseverative reaching by investigating how the direction of experimenter gaze would affect infant search and social behaviour. Infant search and social behaviour was compared on B trials across three different conditions, namely experimenter gaze to midline, location A and location B. Search performance significantly improved when the experimenter looked to location B. Infant social looking indicated that infants expect the object to be found in the location in which they search and are actively seeking information about object location from the experimenter. We conclude that social looking is a valid index of infant expectation that has provided support for the importance of the social environment on the AB search task. This casts doubt on the potential for this task to provide information related to the development of object permanence in infancy.

## Graphical abstract

In comparison to infant search and social behaviour on a standard version of the A‐not‐B search task, when the experimenter directed their gaze towards the correct location on B trials, infants made fewer search errors. When the experimenter directed their gaze towards the incorrect location, infants made more search errors and initiated more social looks. We conclude that understanding the infant's active social behaviour in this task is integral to understanding the A‐not‐B search error.

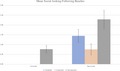

**Research Highlights**

- This paper compares infant search and social behaviour in the AB search task when the experimenter holds a midline, B location or A location gaze.
- When the experimenter looked to location B on B trials, few infants made incorrect searches compared with the standard task where eye gaze remained neutral.
- When the experimenter looked to location A on B trials, infants showed longer error runs and initiated more social looks than alternative eye gaze conditions.
- We propose that our understanding of the infant's active social behaviour in this task is integral to understanding the A‐not‐B search error.

## 1 INTRODUCTION

There is a continuing controversy regarding the origins of object permanence. On the one hand, violation of expectation (VoE) studies suggest that infants have an understanding that objects continue to exist when out of sight, which is reflected in longer accumulated looking to events that violate the object permanence. For instance, Baillargeon (1987) showed that infants as young as 3.5 months looked longer when a drawbridge appeared to move beyond the point where the object placed behind it should have caused an obstruction. In experiments involving two locations, infants as young as 8 months old have shown longer looking when an object is retrieved from an incorrect location (Ahmed & Ruffman, 1998; Baillargeon, DeVos, & Graber, 1989; Baillargeon & Graber, 1988). This suggests that young infants have an understanding of object permanence that extends to keeping track of objects when they are hidden in different locations.

On the other hand, when active searching is required, 9‐month‐olds' knowledge of object permanence appears limited. Although they search correctly when an object is hidden in one location (A), they often perseverate towards the first location when the object is subsequently hidden at a second (B) location (Piaget, 1954). Piaget interpreted infants' search behaviour as indicative of a limited and fragile understanding of object permanence. There thus appears to be a disparity between what looking and reaching measures tell us about infants' expectations regarding hidden objects, with looking measures often interpreted as evidence for understanding of object permanence at an early age, while reaching measures often suggest fragile understanding of objects at 9 months and older.

It is important to establish whether these measures are truly providing contradictory information about infant understanding of objects or whether they reflect the development of alternative capacities. Furthermore, it is not clear whether looking and reaching behaviours are in conflict on the A‐not‐B search task itself. Diamond's (1988) anecdotal observation that on B trials infants often looked to location B before reaching to location A indicated a potential for looking and reaching behaviour to provide contradictory evidence of infant understanding. Her interpretation of this was that 9‐month‐old infants have knowledge of objects that they have the capacity to display through looking measures but not through reaching measures. Supporting empirical evidence is provided by Hofstadter and Reznick (1996) who showed that, following standard A trials, infants who reached on B trials made search errors whereas those who watched an experimenter retrieve the object looked to the correct location. In contrast, Smith, Thelen, Titzer, and McLin (1999) report in a series of studies that infants generally looked where they reached and Bell and Adams (1999) reported no significant difference in performance between comparable looking and reaching versions of the A‐not‐B task. Thus, there is mixed evidence for the existence of contradictions between different measures of infant expectation on this task which further contributes to the difficulty in explaining the A‐not‐B search error.

Interpretation of search task data is made more difficult by methodological differences between studies. In particular, the studies reported above use a multiple‐reversal procedure (e.g. Bell & Adams, 1999; Diamond, 1985; Hofstadter & Reznick, 1996) whereby, following successful B trials, the hiding location is switched back to location A (which is now deemed in the experimenter's eyes to be location B). This adds a level of complexity to the design and likely results in increased demand on additional systems that was not present in the original AB task. This makes meaningful comparisons and interpretations in relation to the infant understanding of objects difficult. For example, Bell and Adams (1999) reported success rates on non‐reversal (A) trials that are much lower than to be expected at approximately 55% compared with most reports at approximately 80%–90% using the standard AB procedure (e.g. Bremner, 1978; Bremner & Bryant, 1977). This difference likely reflects additional difficulty of the reversal version of the task. Thus, comparisons of interpretations that are based on data resulting from the multiple‐reversal to the standard procedure should be made with caution.

Alternative measures to looking and reaching on the first B trial have been employed in an attempt to understand more about what infant behaviour on the AB task is telling us about development. Often, manipulations of the task led to increased accuracy at chance levels on the first B trial which can be difficult to interpret (Wellman, Cross, Bartsch, & Harris, 1987). This could be indicative of either moderate improvement in reaching accuracy or confusion/distraction of the infants leading to random reaching. When comparing B trial search performance under three conditions of varying difficulty, Butterworth (1977) analysed error run. This revealed that where search performance based on the first B trial appeared random at a group level, accurate searchers and inaccurate searchers were persistent in their search choice in subsequent B trials. These results highlight the important contribution error run can make when accurately distinguishing between random searching on the first B trial and performance that actually reflects individual differences in success.

Perhaps though, poor performance in reaching behaviour on the A‐not‐B task is not reflective of a fragile concept of object permanence but is actually the result of an entirely different psychological process. There are numerous explanations of search errors without reference to the infant's knowledge of the physical rules surrounding objects. For example, a short delay between when the object is hidden and the when the infant is subsequently allowed to search appears to be essential for poor search performance. This has led many to propose theories that are related to an underdeveloped memory/representation system. Harris (1974) compared the search performance of 10‐month‐olds on a 0‐s and 5‐s delay version of the search task and found errors were more likely to occur when a delay is introduced. Furthermore, when Diamond (1985) introduced a no‐delay modification to her version of the AB task, performance on the search task improved and older infants were better able to cope with longer delays before performance deteriorated. This cannot be accounted for in Piaget's account, as there is no reason why the presence of a delay would detract from infants' knowledge of objects. Diamond interpreted this as evidence for a combination of memory and inhibition processes as explanation of infants' inability to search correctly on B trials. The infant must have the ability to hold a memory trace for location B throughout the delay period and then use this information to override a stronger memory trace for reaching to location A. This account explains disparities between looking reactions and reaching measures with demand on memory being much lower in the former. It must be noted, though, that Diamond's task likely placed more demand on memory processes than the Piagetian AB search task with multiple reversals of A and B locations and so it is not surprising that reducing memory demands aided performance.

Probably one aspect of the error that is hardest to explain is the fact that it occurs even when the object is uncovered and in full view at the B location (Bremner & Knowles, 1984). This creates considerable difficulty for accounts based in the infant's representation or memory for the hidden object (for instance, Diamond, 1985, 1988). The dynamic systems model, alternatively succeeds in explaining behaviour where memory accounts do not, places the root cause in just about every capacity except for the concept of object permanence (Smith et al., 1999). Assuming the problem is much more complex than simply arising from one root cause, this account explains infant behaviour as a result of bodily interactions with the world. The infant combines information from multiple sources, which are integrated to form a decision on motor‐planning. It highlights the following numerous aspects to this task: infant processing of experimenter behaviour and perceptual aspects of the set‐up, remembering, planning, and reaching are all stages with the capacity to create opportunities for influence on the ‘network’ and thus resulting behaviour of the infant. Under this model, infants succeed on looking tasks due to the absence of manual movement requirement and thus there is an absence of contradictory motor traces of location A when required to search at location B. In short, infants fail when the object is in full view on reaching tasks due to a combination of the over‐activation in the network for reaches to location A and the decay of memory (and thus reducing activation) for observing the action at location B. This is similar to Diamond's account, yet there is scope for many environmentalaspectstoinfluence the weighting of activation for locations in a graded manner, for example, visual cues and experimenter social behaviour that could increase activation for location B relative to location A.

One other explanation for search errors that can account for errors with the object in view with a single root cause is based on the notion that, during A trials, infants come to interpret the repeated hiding and retrieval of the object at A as a cue that A is a location at which objects are to be found (Bremner, 1985). More recently, Topál, Gergely, Miklósi, Erdöhegyi, and Csibra (2008) proposed and tested an explicitly social version of this account. Experimenters generally use eye contact and infant‐directed speech when engaging the infant and Topál et al. (2008) proposed that these communicative cues were used by infants to identify the A location as the place at which to search. In order to explore the effects of these social cues, the authors compared infant search behaviour in the following three conditions: communicative (eye contact, smiling, infant‐directed speech), non‐communicative (the experimenter sat facing 90° away from infant with hands and arms still visible, no eye contact or communication) and non‐social (objects were moved through a curtain so that no part of the experimenter could be seen). While 81% of the infants produced the error in the communicative context, only 48% of infants produced the error in the non‐communicative condition and 41% erred in the non‐social condition. Topál et al. (2008) concluded that the social communication between experimenter and infant has a detrimental effect on search performance; effectively the experimenter's pragmatic cues mislead infants into persistent search at A.

Although the percentage of infants erring on B trials reduced, search errors were still reduced only to chance levels and no analysis of error run is reported, meaning it cannot be established from these data whether infants truly became more accurate, or whether they actually resorted to random searching on B trials when social cues were removed. A closer inspection of Topál et al.'s (2008) procedure makes random searching plausible. In the non‐communicative condition, the experimenter sat facing 90° to the infant's line of sight with arms turned towards the infant in order to hide the object. In addition to removing social communication, this added an element of unusual behaviour on the part of the experimenter that infants are not likely to have witnessed previously. Following Trevarthen's (1977) work, the distressing effects of a still‐face response in parent – infant communication have been well‐documented (for review see Mesman, van Ijzendoorn, & Bakermans‐Kranenburg, 2009). Therefore, the lack of communication and strange behaviour of the experimenter may have led to some distress or at the very least, distraction. This could have reduced attention to the task and increased attention to the experimenter, resulting in random searching. Similarly, the non‐social condition, involving a disembodied hand, is unusual and may have reduced attention to the location of the object. Indeed, in their commentary, Spencer, Dineva and Smith (2009) highlight the importance of establishing whether social communication causes distraction in the A‐not‐B task. In the absence of a clear interpretation of chance‐level performance in these conditions, it is difficult to assess the validity of the effect of social communication on the stage IV search task.

Despite these interpretative problems regarding the supporting evidence, the theory presented by Topál et al. (2008) has the dual advantage of being able to explain errors with the object in view and of being reconcilable with evidence from VoE measures, which suggest early knowledge of object permanence. Specifically, it is possible that the infant correctly perceives or represents the location of the object but is misled by experimenter social cues to search in the wrong place. It is thus important to investigate this social miscuing account further.

Generally, on a broader level, due to looking and reaching data offering opposing conclusions related to where the infant might expect objects to be found, it seems appropriate to take a multiple‐measures approach to the study of behaviour on the A‐not‐B task. One relevant measure of infant expectation of object location is social looking. Social looking is a behaviour that Schaffer (1989) applies to behaviour in which infants initiate mutual reference to an external topic (object). This behaviour is adaptive in that young infants do not have the experience to enable their adequate analysis of ambiguous situations and so following the emotional reactions of trusted others may ensure survival in potentially dangerous environments. An infant's first step in establishing joint attention with another is to look to towards the relevant adult, a behaviour found from 6 months of age (Vaillant‐Molina & Bahrick, 2012; Walden & Ogen, 1988). VoE methods have historically made use of looking time as a measure of cognition, which has been criticized on the basis that this behaviour can be explained by low‐level perceptual preferences (Cohen & Marks, 2002; Haith, 1998). Recently, an alternative measure of infant expectation, and therefore cognition, was introduced by Walden, Kim, McCoy, and Karrass (2007), applying this in Wynn's (1992) numerical VoE procedure. Infants initiated more social looks following an inaccurate numerical outcome than an accurate numerical outcome. Although social looking behaviour could also be interpreted as a response to perceptual novelty, Dunn and Bremner (2017) showed that social looking increases when object identity is violated but not when a novel object is introduced. Thus, it appears that the social looking measure of expectation avoids potential confounding with perceptual novelty preference and presents a suitable measure of infant expectation. The utility of this measure in other infant cognition tasks is currently unknown and social looking could, for the first time, provide a viable and valid tool for measuring infant expectation at the time of taking part in the traditional reaching version of the AB search task, for example social looking following an inaccurate reach could be indicative of an expectation that the object should indeed be found in the searched location. This would provide contradictory evidence to accounts that have scope for the infant to mask knowledge of the object's location through an inability to inhibit habitual reaches (e.g. Diamond, 1985).

This paper reports a further test of social miscuing accounts, using more naturalistic manipulations of experimenter eye gaze, enabling the parameters of the search task to remain much closer to the standard paradigm than previous methodologies that remove social cues entirely. While direct eye gaze is a crucial aspect of pragmatic communication used in Topál et al. (2008), directed eye gaze, by 9 months of age, already efficiently directs the attention to, and improves the processing of, objects in the environment (Hoehl, Reid, Mooney, & Striano, 2008; Parise, Reid, Stets, & Striano, 2008; Reid, Striano, Kaufman, & Johnson, 2004). Here, infant behaviour on the B trials of a standard A‐not‐B task with neutral experimenter eye gaze (ambiguous) is compared to B trials on which the experimenter directs eye gaze either to the B (congruent) or the A (incongruent) location, thereby reducing ambiguity of the social environment but providing accurate or inaccurate information. If infants are paying close attention to the social cues of the experimenter, and pragmatic miscommunication is a strong factor in the cause of search errors, directing experimenter social cues to reduce the ambiguity of what is traditionally neutral gaze should improve (in the case of congruent gaze) or impair (in the case of incongruent gaze) search performance.

In addition, the studies reported here make use of a multiple‐measures approach comparing (a) traditionally reported infant error rate, (b) error run and (c) infant social looking. Analysis of error run will not only facilitate accurate interpretations of group level performance on the first B trial (Butterworth, 1977), but will also establish the persistence of the decision to search incorrectly, potentially providing information on the confidence of infants in their decisions across conditions (Goupil & Kouider, 2016). For the first time, social looking is introduced to the A‐not‐B search task as ameasure of infant expectation.Should this measure prove valid, analysis of social looking initiated by infants following their search decision will provide more information related to infant expectations of object location, potentially independent of their reaching decisions (Dunn & Bremner, 2017).

Social accounts predict that infants should expect the object to be found in their location of choice and that infants should attempt to interpret experimenter behaviour. Thus, this paper compares infant search and social behaviour on B trials undercongruent experimenter eye gaze and incongruent experimenter eye gaze conditions as a test of the predictions that can be made by social miscuing accounts. In comparison to behaviour on a standard A‐not‐B task, error runs should be longer under incongruent eye gaze conditions and shorter for those who do make search errors under congruent experimenter eye gaze conditions, as these cues should encourage accurate searching. Infant social looking should increase following an inaccurate reach in comparison with an accurate reach across conditions as infants' expectations are influenced by cues from the experimenter and thus attempts are likely to be made to seek further information for error correction. These analyses will help to assess the alternative accounts of search errors on these tasks as well as investigating the validity of social looking as a measure of infant expectation on the A‐not‐B search task.

## 2 METHOD

### 2.1 Participants

Based on the sample sizes most commonly reported in previous literature for this task, forty‐eight 9‐month‐old infants (*M* = 277.44, *SD* = 9.72 days, 24 females) were assigned to one of the three conditions; (a) standard, (b) congruent social cues and (c) incongruent social cues.

Prior to recruitment, ethical approval regarding the recruitment, methodology and data handling throughout the study was sought and gained from the Lancaster University Ethics Committee. All infants were recruited through phone calls from a database compiled of those mothers who gave birth at the Lancaster Royal Infirmary who expressed an interest in taking part in psychological research. Four infants participated with their father and the remainder took part with their mothers. The sample was predominantly white, middle class. Data from 19 additional infants could not be used because of technical problems (3) or fussiness (16). Crying or inattention to hiding events defined fussy behaviour. No excluded infant completed any B trials.

### 2.2 Materials

Infants sat in a specially designed A‐not‐B error apparatus that consisted of a comfortable, supportive infant chair that could be pulled closer to, and further from, a table. During object hiding, the infant was positioned 30 cm away from the table. This table (30 cm × 60 cm) held the hiding locations (wells 4 cm deep with lips 1.5 cm above the table surface, 8 cm × 8 cm wide, 18 cm apart edge to edge) that were revealed once the covers were removed. The object consisted of two brightly coloured attached letter links manufactured by ‘Sassy’ http://www.sassybaby.com/products/142/product/1359/option/1359. Covers were made from plain black cloth material (12 cm × 12 cm).

### 2.3 Procedure

All infants underwent familiarization trials, warm‐up trials, A trials and B trials.

#### 2.3.1 Familiarization trials

Infants were first familiarized with the covers. Once the experimenter was satisfied that interest in the covers had reduced, the experimenter took the covers in order to begin the warm‐up trials (as in Bremner, 1978).

#### 2.3.2 Warm‐up trials

Following familiarization trials, infants underwent three warm‐up trials in the central space between the two locations. On the first trial, the object was 50% occluded by the cover, in the second, the object was 75% occluded and in the third, the object was 100% occluded. Practice reaching to the neutral location was encouraged on each trial.

#### 2.3.3 A trials

Following familiarization trials, A trials commenced. The investigator lowered the toy in and out of well A three times while audibly counting in order to ensure the infant was attending to the hiding location. After 1 s, the covers were simultaneously placed over both wells. Following a 5‐s delay, the infant was pulled towards the table and was given the opportunity to search for the toy. There were five A trials. The location of the A trials (left or right) was counterbalanced. The investigator maintained central eye gaze in all conditions.

#### 2.3.4 B trials

B trials followed A trials. The investigator lowered the toy in and out of well B three times while audibly counting. After 1 s, the covers were placed over the wells at the same time. Following a 5‐s delay, the infant was brought towards the table and was given the opportunity to search for the toy. In the standard condition, the investigator behaviour continued to maintain central eye gaze. In the congruent and incongruent gaze conditions, the experimenter directed eye gaze to the B and A locations respectively from the time that the object was hidden, throughout the delay and during the search phase of the task. The experimenter aimed to repeat B trials until the infant had either reached correctly on two trials, or had become inattentive to hiding events so that two correct trials could not be attempted. The number of B trials was not fixed so that the error run could be recorded.

## 3 RESULTS

Infant behaviour was coded from video recordings that included three synchronized viewing angles on a split screen. Viewing angles included (a) 45° infant line of sight, left hand‐side view of infant, caregiver and experimenter, (b) 45° infant line of sight, right‐hand view of infant, caregiver and experimenter and (c) 45° experimenter line of sight, right‐hand view of experimenter and caregiver. Manual search was measured in terms of accuracy on the first B trial versus the final A trial, and the number of A and B trials on which the infant searched incorrectly before the correct location was chosen (error run). Interobserver reliability, calculated for 25% of participants, was high (intraclass correlation coefficients [ICCs] = 0.836 and 0.922 for social looks following accurate and inaccurate reaches, respectively).

### 3.1 Search accuracy

As Table 1 shows, in the standard condition, there was a significant overall change in the search accuracy from A to B trials, McNemar χ^2^ (1, *N* = 16) = 10.08, *p* = .0015. In the congruent gaze condition, there was a significant overall change in the search accuracy from A to B trials, χ^2^ (1, *N* = 16) = 5.818, *p* = .016. In the incongruent gaze condition, there was a significant overall change in the search accuracy from A to B trials, χ^2^ (1, *N* = 16) = 6.750, *p* = .009.

**Table 1:** Search accuracy on the final A trial and the first B trial across standard, congruent and incongruent gaze conditions

	Standard condition		Congruent gaze		Incongruent gaze	
	A correct	A incorrect	A correct	A incorrect	A correct	A incorrect
B correct	3.00	0.00	5.00	1.00	3.00	1.00
B incorrect	12.00	1.00	10.00	0.00	11.00	1.00

### 3.2 Error rate

Infants took part in an average of 3.81 (range: 2–7), 2.94 (range: 2–5) and 4.31 (range: 2–9) B trials in the standard, congruent and incongruent conditions respectively. Mann – Whitney U tests were used to determine the differences in error run between groups based on gender and A location (left or right) on A and B trials. No significant differences were found.

**Figure 1:**
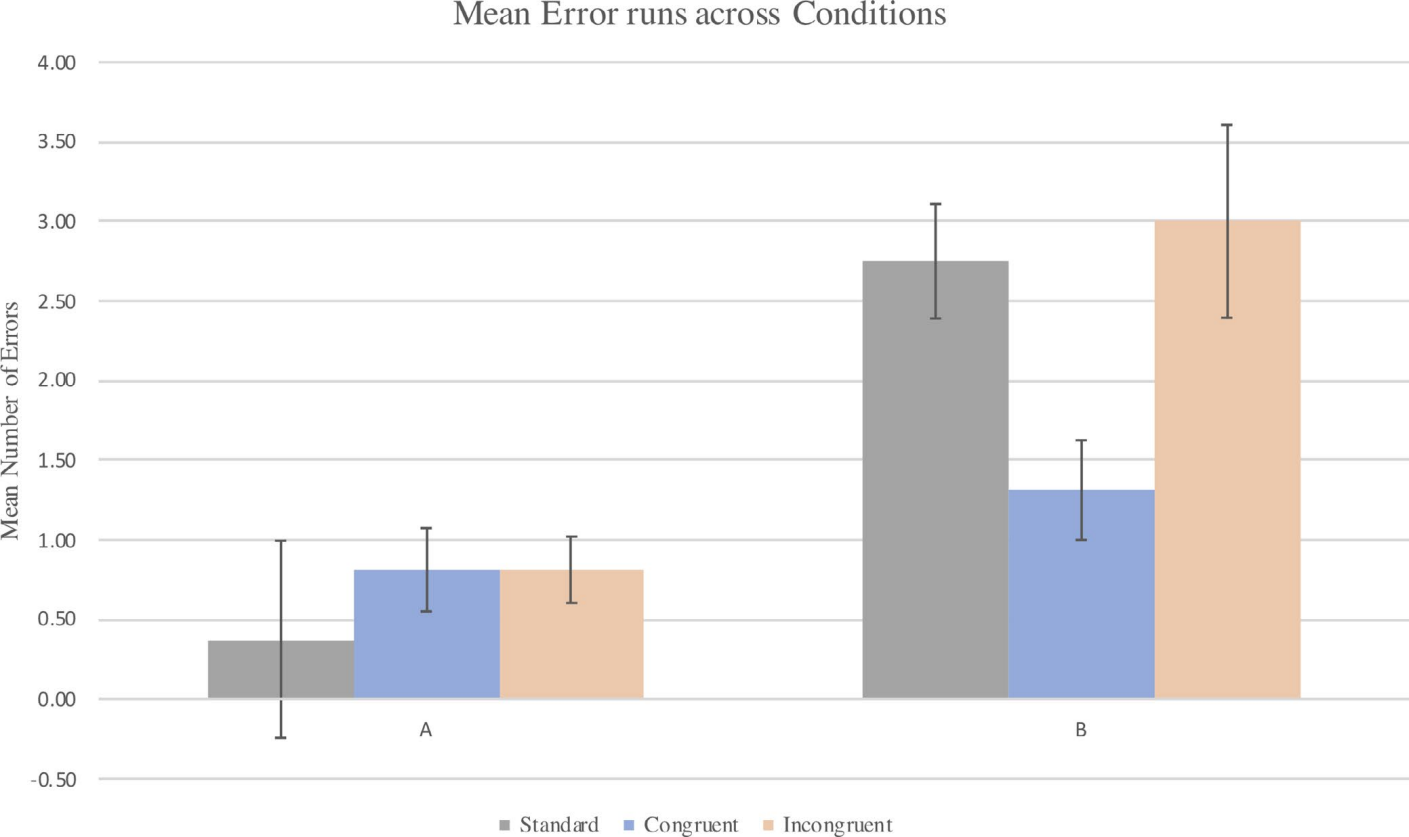
The mean length of error run on A and B trials for those in the standard, congruent and incongruent social cue conditions. Error bars represent standard error

A series of Wilcoxon signed‐rank tests were used to investigate the differences in error run on A and B trials within each condition. Figure 1 shows that for the standard condition, infants made more errors on B trials (*M* = 2.75, *SD* = 1.44) than A trials (*M* = 0.38, *SD* = 0.62), *Z* = 3.562, *p* = <.001. In the incongruent gaze condition, infants also made more errors on B trials (*M* = 3.0, *SD* = 2.42) than A trials (*M* = 0.81, *SD* = 0.83), *Z* = 2.488, *p* = .013. There was no significant difference in errors made between A trials (*M* = 0.81, *SD* = 1.05) and B trials (*M* = 1.31, *SD* = 1.25) for those in the congruent gaze condition, *Z* = 0.996, *p* = .319.

A Kruskal – Wallis test was used to investigate the differences in error run on A and B trials for infants in the standard, congruent and incongruent gaze conditions. There was no significant difference between conditions in the number of errors made on A trials, χ^2^ (1, *N* = 48) = 2.734, *p* = .255. There was a significant difference in the number of errors made between conditions on B trials only, χ^2^ (1, *N* = 48) = 9.147, *p* = .01. Further analysis using a series of Mann – Whitney U tests revealed no significant difference in errors on B trials between standard (*M* = 2.75, *SD* = 1.44) and incongruent gaze conditions (*M* = 3.0, *SD* = 2.42), *U* = 127.5, *p* = .98. There were significantly fewer errors on B trials in the congruent gaze condition (*M* = 1.31, *SD* = 1.25) than the standard condition (*M* = 2.75, *SD* = 1.44), *U* = 55.5, *p* = .005. Likewise, there were significantly fewer errors on B trials in the congruent gaze condition (*M* = 1.31, *SD* = 1.25) than the incongruent gaze condition (*M* = 3.0, *SD* = 2.42), *U* = 66.0, *p* = .017.

### 3.3 Social looking

Infants' social looking was coded from video recordings following a search decision until the object was found and in the following 5 s. Due to the multiple viewing angles of the recordings, the direction of infant looking could be clearly recognized. Multiple looks to the experimenter contributed to a score. For instance, should an infant look to the experimenter twice within a given trial, the social looking score for that trial would be 2. Owing to the small number of inaccurate searches on A trials, social looking behaviour following accurate and inaccurate reaches was analysed for B trials only. As the number of B trials was not fixed, social looking was calculated as a proportion by dividing the number of social looks coded by the number of trials an infant performed. Mann – Whitney U tests were used to determine the differences in social looking between groups based on gender and A location (left or right). No significant differences were found.

**Figure 2:**
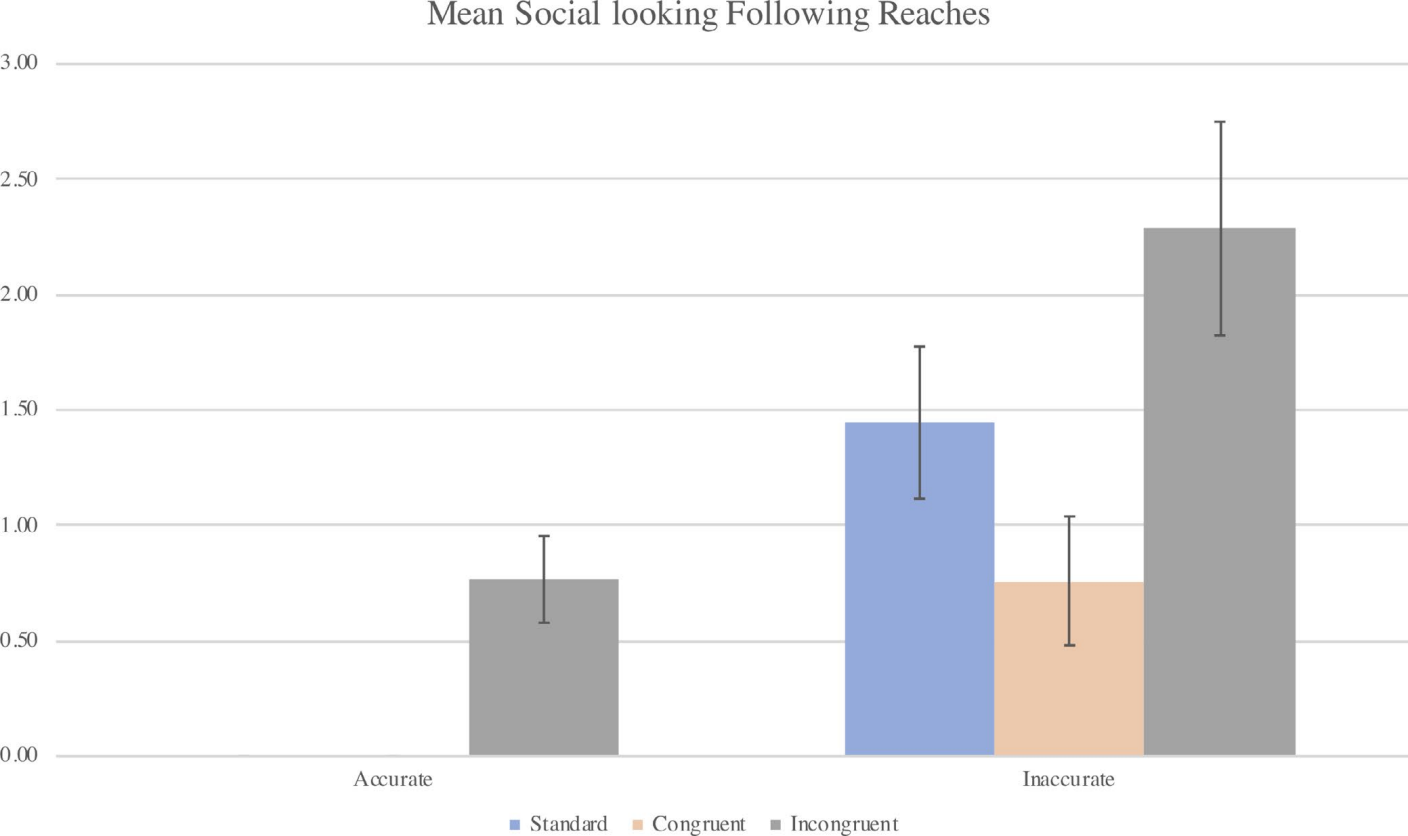
The mean proportion of social looks initiated following accurate and inaccurate reaches on B trials for those in the standard, congruent and incongruent social cue conditions. Error bars represent standard error

A series of Wilcoxon signed‐rank tests were used to investigate the differences in social looking on B trials following accurate and inaccurate reaches within each condition. Figure 2 shows that for the standard condition, infants initiated more social looks following an inaccurate reach (*M* = 1.45, *SD* = 1.32) than an accurate reach (*M* = 0.00, *SD* = 0.00), *Z* = 2.941, *p* = .003. In the congruent gaze condition, infants also initiated more social looks following an inaccurate reach (*M* = 0.76, *SD* = 1.12) than an accurate reach (*M* = 0.00, *SD* = 0.00), *Z* = 2.375, *p* = .018. Likewise, infants in the incongruent gaze condition initiated more social looks following an inaccurate reach (*M* = 2.29, *SD* = 1.85) than an accurate reach (*M* = 0.77, *SD* = 0.73), *Z* = 2.238, *p* = .025.

A Kruskal – Wallis test was used to investigate the differences in social looking on B trials between conditions for infants in the standard, congruent and incongruent gaze conditions. There was a significant difference in social looking between conditions following accurate reaches, χ^2^ (1, *N* = 48) = 19.860, *p* = <.001, and inaccurate reaches, χ^2^ (1, *N* = 48) = 7.710, *p* = .021. Further analysis using a series of Mann – Whitney U tests revealed more social looking following inaccurate reaches on B trials in the standard (*M* = 1.45, *SD* = 1.31) than the congruent gaze condition (*M* = 0.76, *SD* = 1.12), U = 76.5, *p* = .048. Likewise, significantly more social looking occurred following inaccurate reaches in the incongruent gaze condition (*M* = 2.29, *SD* = 1.85) than the congruent gaze condition (*M* = 0.76, *SD* = 1.12), U = 63.5, *p* = .013.

Significantly more social looking occurred following accurate reaches in the incongruent gaze condition (*M* = 0.77, *SD* = 0.73) than in the standard condition (*M* = 0.00, *SD* = 0.00), U = 36, *p* = .002. In addition, more social looking occurred following accurate reaches in the incongruent gaze condition (*M* = 0.77, *SD* = 0.73) that in congruent condition (*M* = 0.00, *SD* = 0.00), U = 45, *p* = .001. No further comparisons were found to be significant.

## 4 DISCUSSION

These results show a clear effect of experimenter eye gaze on infant search and social behaviour (see Table 2 for a summary of behaviour with interpretation). In the standard condition where the experimenter held their gaze to the midline, the A‐not‐B error occurred, with infants making more search errors and showing longer error runs on B trials than A trials. On B trials, when the experimenter looked to location B, infants showed significantly better search performance than when the experimenter looked to the midline or location A.

**Table 2:** Search performance and proportion of social looks to the experimenter on B trials with authors' interpretation of infant's social looking behaviour

Condition (experimenter eye gaze during the B trial delays)	Group level of search performance on B trials	Frequency of social looks to the experimenter on B trials (and implied child expectation) after:	
		Correct searches (B)	Incorrect searches (A)
Directed towards the B location (congruent looking)	At chance, but not random; children switched to B quickly	No looks (high expectation it will be at B)	Low frequency (low expectation it will be at A)
Remained neutral (standard A not‐B task)	Perseverative searches to A	No looks (high expectation it will be at B)	Medium frequency (medium expectation it will be at A)
Directed towards the A location (incongruent looking)	Perseverative searches to A	Low frequency (lower expectation it will be at B; expectation that adult provides helpful information)	High frequency (high expectation it will be at A; expectation that adult provides helpful information)

In comparison to experimenter looking to the midline, search behaviour significantly improved when the experimenter looked to location B but did not deteriorate as a result of experimenter looking to location A. This suggests congruent social cues are more likely to have an effect on search performance than incongruent cues. This could have been due to a ceiling effect, given that the error rate in the midline condition was already relatively high (81%). Furthermore, many infants in all conditions became fussy after 3 to 4 B trials and so it was often impossible to determine whether incorrectly reinforcing cues would have led to a longer error run. Thus, a lack of a significant deterioration in performance must be interpreted with caution.

Infants made fewest B trial errors when the experimenter looked towards location B and showed most social looking when the experimenter looked to the A location. Thus, infants seek, interpret and follow experimenter eye gaze during the search task and show a particular response suggestive of checking when that eye gaze does not match the object's location. Consequently, consistent strong support was found across measures for the inclusion of the social environment in accounting for perseverative errors. This limits the contribution that behaviour in these tasks can make towards our understanding of the development of object concept in infancy (Piaget, 1954). Alternative accounts of perseverative reaching also fail to predict the moderate improvement in search performance on the first B trial shown in the presence of congruent social cues. Not only do memory accounts (e.g. Diamond, 1985; Harris, 1974) fail to predict errors while the object is in full view of the infant (Bremner & Knowles, 1984), they predict a larger improvement in search performance than is reported here (assuming cues to the congruent location lessen the demand placed on memory for the search location). The dynamic systems model (Smith et al., 1999) might best explain moderate improvement on the first B trial and a shorter error run as this allows for a graded response to social cues. Although congruent eye gaze cues might reduce the demand on memory for the hiding event at location B, it might still be difficult to inhibit/override the memory trace for reaching to location A. Thus, reaches on the first B trial might still be affected by previous reaches to the A location. Following this trial, congruent cues might enable the memory trace for location B to strengthen more quickly than in the standard condition leading to the shorter error runs found in this paper.

Perhaps, though, there is more moderate support than would be expected for Topál et al.'s (2008) account, which identified a pragmatic misinterpretation of ambiguous social cues given by the experimenter as the sole source of the AB error. Arguably, very subtle changes in social cues (the movement of the experimenter eye gaze) influence the search performance. Thus, our results show that infants are highly attentive to the social behaviour of the experimenter during the delay period, a period when they might plausibly be focussing attention primarily on the location of the recently hidden attractive object. Under the assumption that directing eye gaze provides disambiguation of social cues, many of the predictions of the pragmatic misinterpretation account are supported by the current results. Directing social cues, as predicted by this account, led to a moderate reduced error on the first B trial just as the removal of social cues did in the Topál et al. (2008) study. In the context of one trial with two location choices, a moderate reduction in error could be explained by either random reaching for all infants or an increase in the ability of a proportion of infants to complete the task. Here, the additional measure of error run (Butterworth, 1977) makes it possible to ascertain whether searching was random. Error run provides information related to the consistency of the error and, potentially, confidence in search decision (Goupil & Kouider, 2016). Error run data suggest that moderate responding on the first B trial was due to better performance rather than random reaching, because there were consistently shorter error runs for those who did err when eye gaze was directed to the congruent location. Thus, congruent social cues reduced the number of infants who made errors and helped those who did make errors to correct themselves faster than those who were given incongruent cues. Yet one question remains. Although search performance improved with the use of helpful experimenter social cues, if the misinterpretation of experimenter behaviour is the sole cause of search errors, why is search not improved to better levels on the first B trial here, or indeed in other studies that manipulate social aspects of the task (Boyer, Pan, & Bertenthal, 2011; Topál et al., 2008)?

For the first time, social looking behaviour (Dunn & Bremner, 2017; Walden et al., 2007) was harnessed as a measure of infant expectation on the A‐not‐B search task and its validity was shown across three different conditions. Infants initiated more social looks following an inaccurate reach than an accurate reach. This cannot be explained by the suppression of social looking due to the allocation of attention to the toy that is revealed on accurate reaches. Infants engaged in more social looking when the experimenter looked to the incongruent location even when the toy was found. The authors interpret behaviour across these conditions to be reflective of an expectation that the object should be found in the reached‐for location. Interpretation of this measure contrasts with those who conclude on the basis of looking time measures that infants actually expect the object to be in the correct location even when they reach to the incorrect location (Ahmed & Ruffman, 1998; Baillargeon et al., 1989; Baillargeon & Graber, 1988; Hofstadter & Reznick, 1996). However, there may be reason to question overall looking is a valid measure of their expectation (Dunn & Bremner, 2017). Furthermore, no previous study has measured expectation on a task that involves the full criteria of the A‐not‐B search task (hidden object, active search). The current study measures infant expectation during active search for a hidden object when social cues are given and social looking has a provided a valid and useful measure of infant expectation.

Social looking behaviour has provided strong support for the role of the social environment in perseveration. Social looking only increased following an accurate reach to location B when the experimenter was incorrectly looking to location A. This could be explained by a violation of infant expectation that an adult should provide helpful information. Although the pragmatic misinterpretation model could account for this on the basis of infants' close attention to social cues, the dynamic systems model (with the inclusive element of active decision‐planning) is arguably best able to explain this result. In this model, infants would expect the object to be found in their reached‐for location and should be surprised (and thus increase social looking) when their reach is accurate and the adult is providing inaccurate information. As the first paper to report social looking as a measure of expectation on the A‐not‐B task, it is important to further investigate this interesting result.

By taking a multiple‐measures approach, our results have revealed an important set of findings relating to the development of object permanence. Error run analysis revealed a moderate, yet not random, improvement in performance in the presence of congruent social cues. Analysis of social looking behaviour revealed information‐seeking behaviour following incorrect searches. Clearly infants are heavily engaged in social communication with the experimenter throughout the task and so it is likely that the social environment plays a strong role in perseverative reaches. However, without evidence of a stronger influence of congruent cues on search performance, it is difficult to rely on pragmatic misinterpretation of traditionally ambiguous cues as a sole cause of search errors. Likewise, behaviour on this task is unlikely to solely reflect infants' understanding of objects if at all. This leaves the dynamic systems theory best able to account for the outcome of the multiple measures presented in this paper. Although social communication clearly has a crucial role, behaviour in this task is likely the result of a process that is too complex for there to be a single cause of search errors.

## CONFLICT OF INTEREST

The authors have no conflict of interests to declare.

## Data Availability

The data that support the findings of this study are available from the corresponding author upon request.
